# A tumor targeting oncolytic adenovirus can improve therapeutic outcomes in chemotherapy resistant metastatic human breast carcinoma

**DOI:** 10.1038/s41598-019-43668-8

**Published:** 2019-05-16

**Authors:** Ali Sakhawat, Ling Ma, Tahir Muhammad, Aamir Ali Khan, Xuechai Chen, Yinghui Huang

**Affiliations:** College of Life Science and Bioengineering, Beijing University of Technology, 100 Ping Le Yuan, Chaoyang, 100124 Beijing China

**Keywords:** Cancer therapeutic resistance, Targeted therapies

## Abstract

Breast cancer is the most prevalent malignancy in women, which remains untreatable once metastatic. The treatment of advanced breast cancer is restricted due to chemotherapy resistance. We previously investigated anti-cancer potential of a tumor selective oncolytic adenovirus along with cisplatin in three lung cancer cells; A549, H292, and H661, and found it very efficient. To our surprise, this virotherapy showed remarkable cytotoxicity to chemo-resistant cancer cells. Here, we extended our investigation by using two breast cancer cells and their resistant sublines to further validate CRAd’s anti-resistance properties. Results of *in vitro* and *in vivo* analyses recapitulated the similar anti-tumor potential of CRAd. Based on the molecular analysis through qPCR and western blotting, we suggest upregulation of coxsackievirus-adenovirus receptor (CAR) as a selective vulnerability of chemotherapy-resistant tumors. CAR knockdown and overexpression experiments established its important involvement in the success of CRAd-induced tumor inhibition. Additionally, through transwell migration assay we demonstrate that CRAd might have anti-metastatic properties. Mechanistic analysis show that CRAd pre-treatment could reverse epithelial to mesenchymal transition in breast cancer cells, which needs further verification. These insights may prove to be a timely opportunity for the application of CRAd in recurrent drug-resistant cancers.

## Introduction

Breast cancer is the most prevalent malignancy in women, which remains untreatable once metastatic^1^. The most commonly practiced method to treat breast and ovarian cancers involve combination therapy integrating radiotherapy or surgery with chemotherapy. However, the severe impediment posed to this treatment strategy is the development of multidrug resistance (MDR) which undermines the effectiveness of cisplatin or other chemotherapy drugs. MDR is among the prominent factors due to which most of the patients receive an early relapse of higher rate after successful initial treatment with chemotherapy^2–6^. The patients expressing MDR are left with very few treatment choices. In such cases, significant therapeutic responses can only be obtained at very high chemotherapy doses, which culminate in serious side effects including, but not limited to myelosuppression, gastrointestinal disorder, and also lifelong cardiac and renal consequences^7,8^.

Regardless of numerous recent advances in cancer treatment, MDR impedes positive therapeutic outcomes of chemotherapy in many cancers of adults and pediatric^9–12^. MDR subsides or abolishes the potency of chemotherapy by overexpressing drug efflux transporters belonging to ATP binding cassette (ABC) family on tumor surface, which decrease the intracellular retention of numerous anticancer agents to sub-therapeutic levels. P-glycoprotein (P-gp/ABCB1) is the most extensively explored ABC family drug efflux pump encoded by the MDR1 gene^13,14^. The overexpression P-gp/ABCB1 leads to resistance towards many currently available chemotherapeutics^15^. Therefore, this is a high priority area in cancer research which demands extensive investigations to find a new strategy or a drug agent which have the potential to deal with the MDR-related inefficiency of chemotherapeutic drugs. A therapy, which combines low doses of DDP or such drugs with other available anti-cancer approaches, could prove a promising strategy to maintain therapeutic effectivity of chemo-agents and to minimize toxicity towards normal tissues^16^.

Cancer gene therapy offers many unique combination opportunities with other available anti-cancer approaches^17^. The engineering of viral vectors, to develop such combination therapies, which have potential to synergize with the virus and promote safety and efficacy of antitumor agents, is still the most important strategy for cancer gene therapy. Oncolytic Adenoviruses (OAs), by virtue of their high transduction potential in both proliferating and quiescent cells, high titers and the capacity to integrate with large cytotoxic transgenes, have become an intensely investigated and potent anti-tumor agents^18–20^. The antitumor efficacy of OA’s, among other factors, is also largely dependent on the coxsackievirus/adenovirus receptor (CAR) whose higher expression on tumor cells is much needed for their entry into these cells^21^. Considering a higher expression of survivin (inhibitor of apoptosis protein) in tumors as compared to healthy cells, it has been investigated for use in gene therapy to acquire tumor specificity^22,23^. Cancer-specific Chemo-Gene-Viro-Therapy (CGVT) involving cisplatin or DDP and a genetically modified conditionally replicating OA, could provide cancer targeted synergistic inhibition and may sensitize resistant tumors to chemo-agents^24–26^.

In our previous work, we engineered and tested the anti-tumor potential of a survivin regulated CRAd in lung cancer cells and demonstrated that its co-administration with cisplatin considerably improved cancer-killing efficacy in a synergistic manner. RT-PCR analysis showed that the expression of Coxsackievirus and adenovirus receptor (CAR) was enhanced by cisplatin which increases the CRAd transduction in cancer cells. CRAd exhibits high tumor specificity owing to cancer-specific replication regulated by survivin and raised CAR expression. To our surprise, CRAd monotherapy displayed strong toxicity towards cisplatin-resistant lung cancer cells^27^. In the current study, we extended our investigation of using Survivin Responsive CRAd to chemotherapy-resistant cancers of different origin. We employed drug resistant breast cancer cells to evaluate whether the applicability of observed outcomes was cancer type specific or whether it could be accepted more generally.

## Results

### Resistance in breast cancer cells and upregulation of CAR

**Figure 1:**
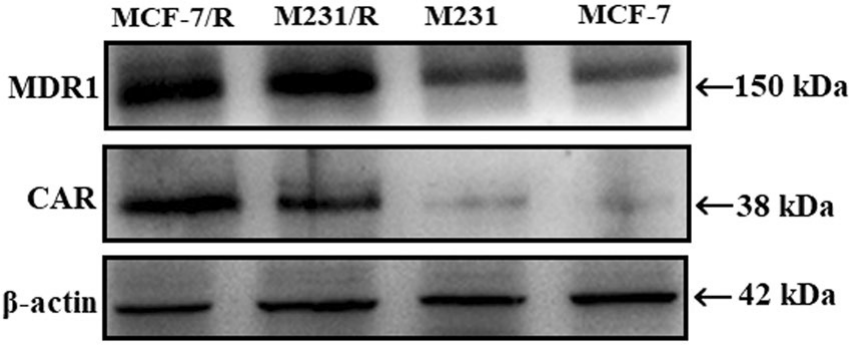
MDR and CAR status in cells. The cell extracts were analyzed for expression of CAR and MDR1 protein. Western blotting was performed using anti-mdr1 and anti-CAR antibodies. Results showed overexpression of both CAR and MDR1 proteins in chemotherapeutic resistant cell lines of both breast cancer cells. Blot images were cropped for clarity of the presentation. Uncropped blots are shown in the supplementary information.

Semi-quantitative RT-PCR was first performed to check mRNA levels of four important members of ABC transporters family and, CAR. Only one of the ABC transporters family member, ABCB1, and CAR were found upregulated at mRNA levels in chemo-resistant sublines of breast cancer cells (data not shown). Subsequently, western blot analysis of upregulated genes was carried out with the intent to confirm and evaluate the resistance and its cause in chemotherapy-resistant breast cancer cells. Higher protein expressions of MDR1, and CAR confirmed the overexpression of these genes in MCF-7/R and M-231/R cancer cells (Fig. 1).

### Raised efficacy of Adv transduction

**Figure 2:**
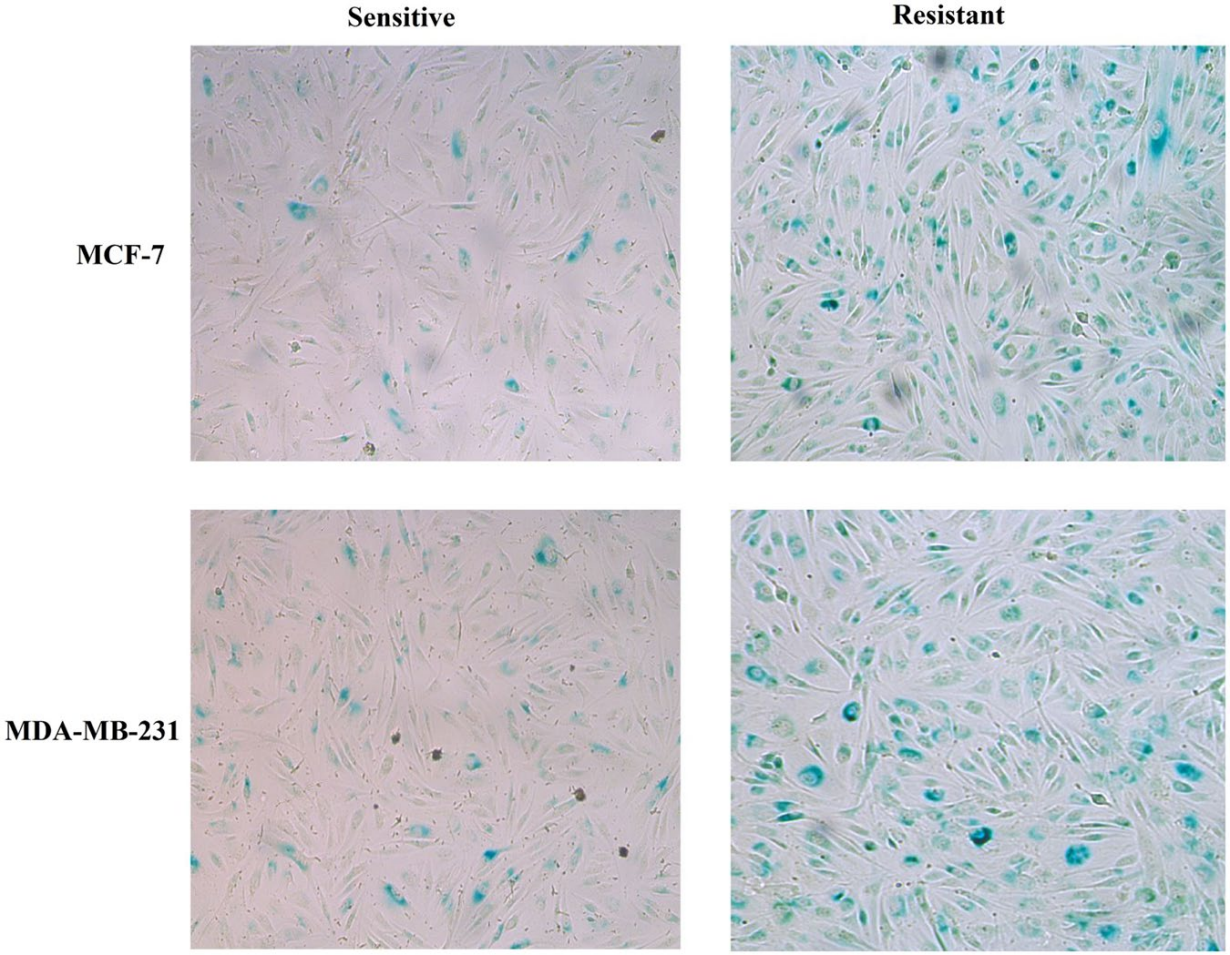
Adenoviral infection efficacy. Both breast cancer cell lines and their sublines were assayed adenoviral efficacy via X-gal staining. Breast cancer cells cultured in six-well plates were treated with different doses of Adβgal, and after 48 hours washed, fixed and stained. Resistant cells exhibit higher rated of transduction.

**Figure 3:**
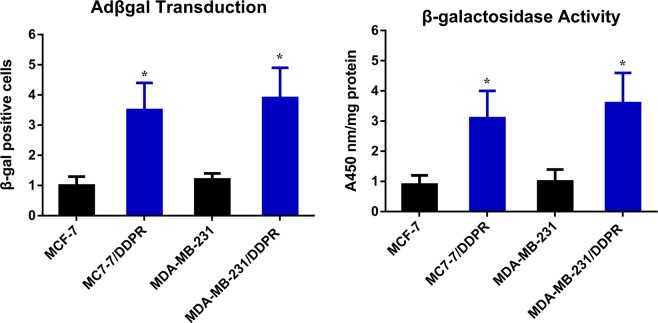
Quantitative β-gal activity measurement. 10^6^ cells were cultured in a 90 mm dish for 2 days,infected for two hours with Adβgal at 4 MOL, lysed 48 hours later to measure β-galactosidase activity and normalized to the amount of protein in each sample determined by Bradford method. Transduction activity in resistant cells of both cell lines is shown relative to sensitive cells of both cell line. Significance was evaluated by two –tailed student’s test in graphed prism6 (*p < 0.05).

To evaluate adenoviral infection efficacy in both cell lines and their sublines, X-gal staining was performed. Though the respective infectability indicated by X-gal staining of both cell lines was variable, the significant difference was observed among chemotherapy sensitive and resistant cells. Cisplatin sensitive and resistant cells of both cell lines were infected with Adβgal at 4 MOI. 48 hours later, the cells expressing the bacterial β-galactosidase gene were observed. All cells infected with Adv exhibit positive blue pigment, but resistant cells showed nearly 4-folds higher number of positive cells (Blue) than sensitive cells. Similar results were obtained upon β-galactosidase activity measurement using Bradford assay (Figs 2 and 3).

### Overexpression of MDR1 and CAR genes increases CRAd transduction which reduces viability in MDR phenotypes of Breast cancer cells

**Figure 4:**
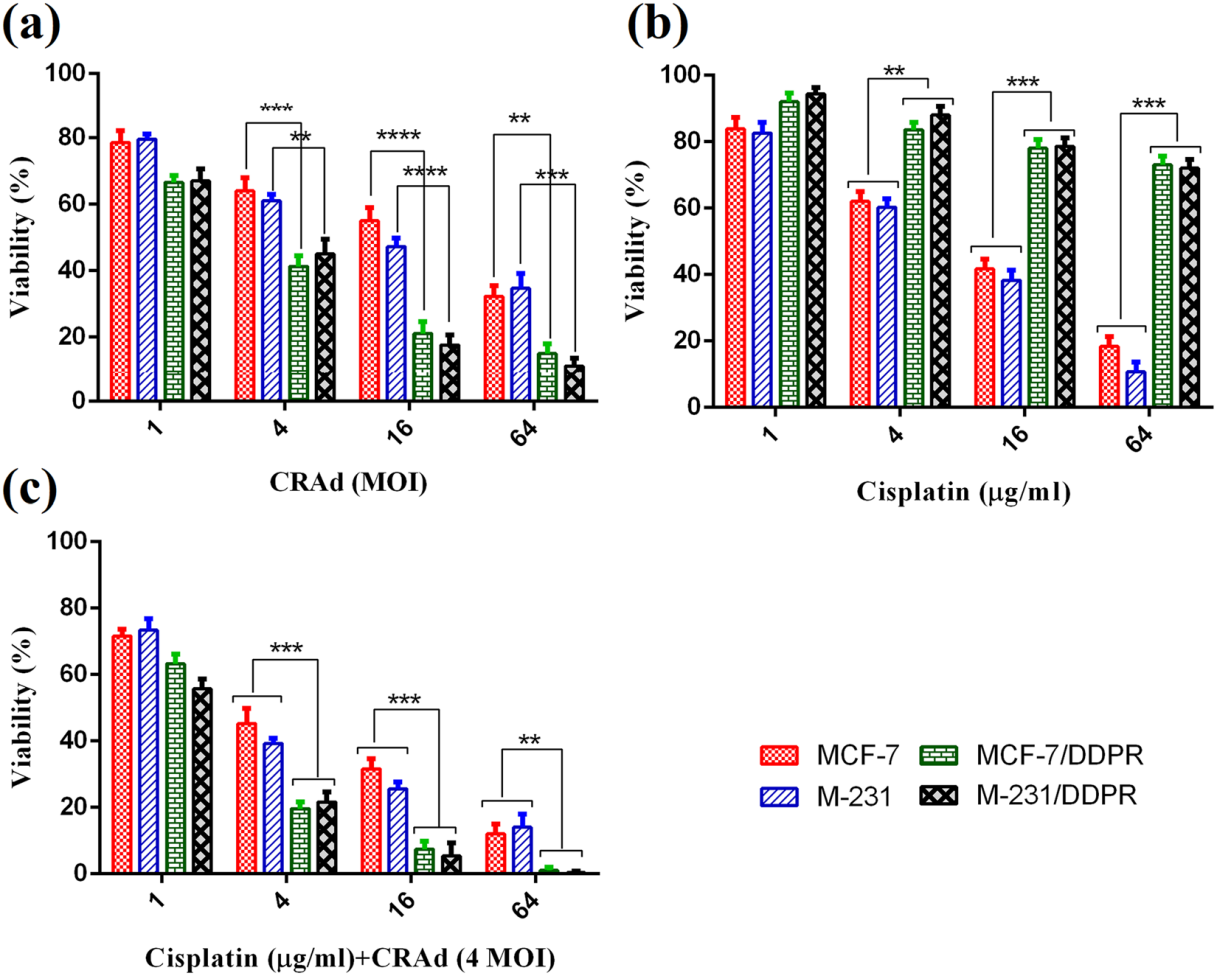
Tumor cell viability analysis via MTT assay. (**a**) Enhanced sensitivity of breast cancer phenotypes towards CRAd. Both breast cancer cell lines were treated with CRAd at different MOIs (1–64). Chemotherapy-resistant cells of both cell lines exhibited significantly high inhibition rates. (**b**) Sensitivity of breast cancer cells towards cisplatin. Both phenotypes of breast cancer cell lines showed a tremendous difference in response to variable concentrations of cisplatin. MCF-7/DDPR and M-231/DDPR exhibit very less sensitivity due to resistance. (**c**) Inhibitory effects of combined treatment with different cisplatin concentrations. High initial inhibition rate was achieved even at a lower dose of cisplatin, and a continuous increase in inhibition was seen with increasing cisplatin doses. The data shown are the average of triplicate experiments. Data are presented as the mean ± SD in A, B and C, (n = 3), *p < 0.05, **p < 0.01, ***p < 0.001, ****p < 0.001 by two-tailed Student’s t test.

*In vitro* analysis performed through MTT assay revealed that upon CRAd infection, the decrease in percent cell viability in MDR phenotype cancer cells (MCF-7/DDPR, M-231/DDPR) was very significant as compared to that was observed in chemotherapy-sensitive cells. The decrease in cell viability was directly correlated with multiplicity of infection of CRAd. At 4 MOI, ≤45% viability was observed in resistant breast cancer cells of MCF-7/DDPR and M-231/DDPR. The relative viabilities in chemotherapy-sensitive cells (MCF-7 and M-231) were ≤65% (Fig. 4a). These results are similar to those were obtained in our previous study with lung cancer cells (A-549 and A-549/DDPR)^27,28^. The molecular mechanism behind this significant difference in viabilities between chemo-sensitive and resistant cells is the enhanced CAR expression which increases viral transduction and subsequent oncolysis.

### Prior exposure to cisplatin sensitizes breast cancer cells to CRAd infection

Chemotherapy-sensitive cells of breast cancer were treated with cisplatin concentrations as described earlier^27^. The dose of cisplatin varies from 1–64 µg/ml. After 4–6 hours following cisplatin treatment, CRAd was added to each treatment group at 4 MOI. Cell mortality was measured performing MTT assay. The data of the assay indicated relatively more reduction in the viability of cancer cells of both cell lines. It confirms the hypothesis from our previous study that prior exposure to cisplatin can increase the viral transduction and hence the cell death in dose-dependent manner (Fig. 4b). Figure 1b indicates that the monotherapy of cisplatin also resulted in higher cytotoxicity and cell death at higher doses but in combination with CRAd, it is very useful in reducing the cell viability in more or less synergistic manner at even lower dose of cisplatin, 4 µg/ml (Fig. 4c).

### Impact of CAR expression on viability of breast carcinoma cells

**Figure 5:**
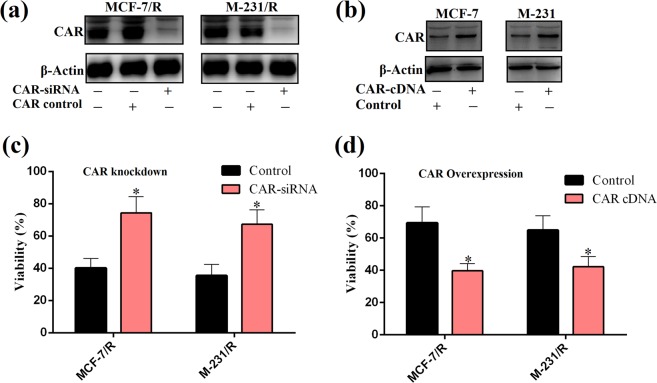
Correlation of CAR expression and CRAd-induced tumor inhibition in breast cancer cell lines. (**a**) CAR inhibition via stable transfection of CAR-siRNA diminished its protein expression in cisplatin resistant cells. (**b**) Ectopic CAR expression through full-length human CAR cDNA raised CAR protein levels in cisplatin sensitive cells. (**c**) CAR knockdown in resistant cells, MCF-7/R and M-231/R significantly reduces the CRAd-induced tumor inhibition. (**d**) CAR overexpression significantly increases the CRAd-induced tumor inhibition in sensitive cells, MCF-7 and M-231. The data shown in (**c**,**d**) are the average of triplicate experiments. Data are presented as the mean ± SD (n = 3), *p < 0.05 by two-tailed Student’s t test. Uncropped blots are shown in the supplementary information (See Supplementary Data, Fig. S3).

To evaluate the influence of CAR on breast cancer biology, we chose chemotherapy resistant cells (MCF-7/R and M-231/R) for CAR downregulation, and sensitive cells (MCF-7 and M-231) for CAR over expression. Western blotting confirmed that CAR expression was reduced in both resistant cells when transfected with CAR-specific siRNA, whereas ectopic expression of pEGFP-N1cDNA in MCF-7 and M-231 cells resulted in a noticeable rise of CAR protein levels (Fig. 5a,b). Next, we evaluated the impact of CAR downregulation and overexpression on anti-tumor potential of CRAd via MTT assay. We observed that anti-proliferative efficacy of CRAd was reduced in chemotherapy resistant cells which might be due to reduced viral infection resulted from CAR downregulation (Fig. 5c). On contrary, CRAd treatment reduces cell viability in chemo-sensitive cell lines with ectopic CAR overexpression (Fig. 5d). It can be speculated from these experiments that the success of virotherapy involving CRAd is linked to CAR expression which is usually low on tumor surfaces, but could be high in resistant cases and in the instances where cancer cells are pre-treated with cisplatin or CAR cDNA containing vector.

### Migration of multidrug-resistant cells of the breast was inhibited by CRAd

**Figure 6:**
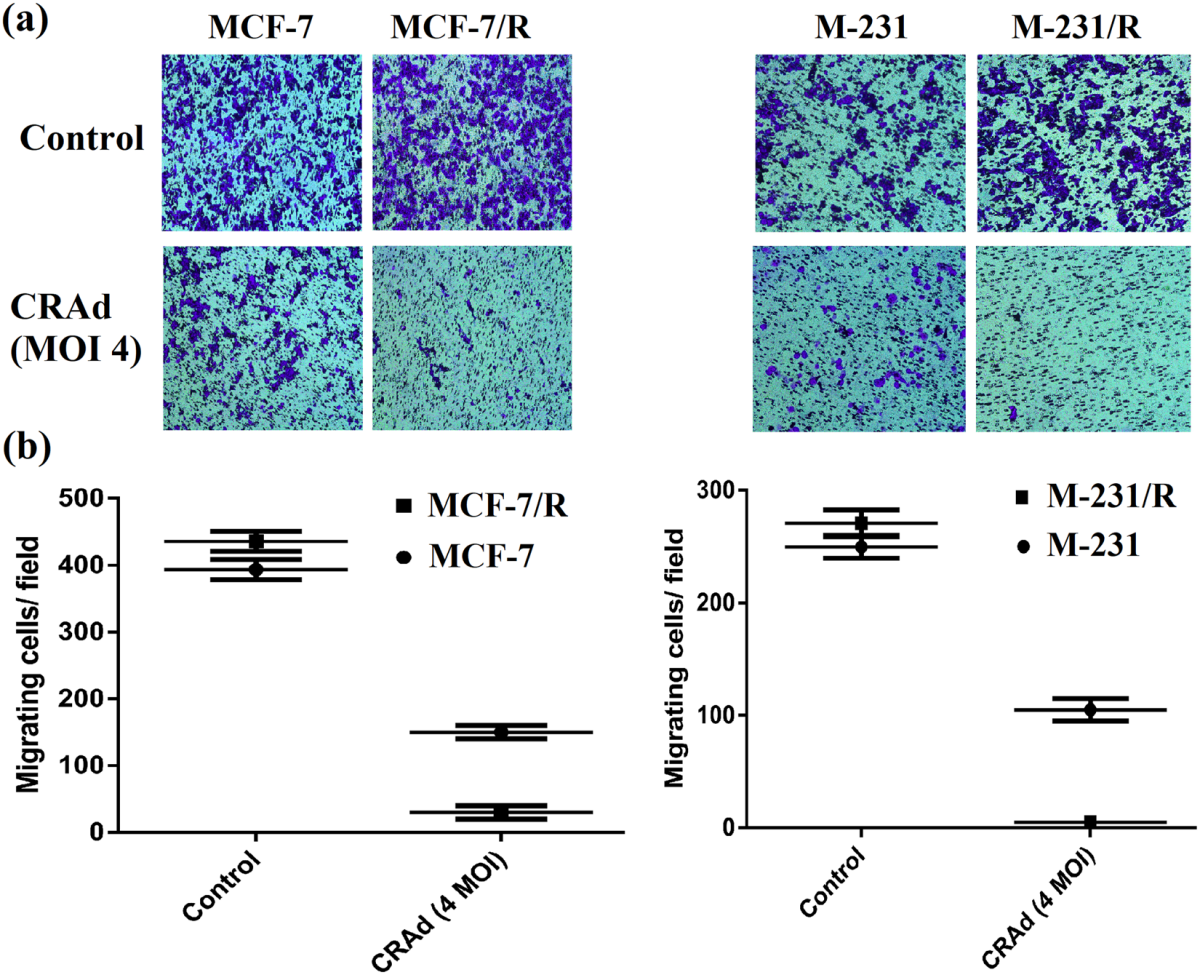
Tumor cell migration study via transwell assay. (**a**) Micrographs taken through phase contrast microscope (under 40x objective, at 100x magnification) showing migrated cancer cells. Crystal violet (0.5%) stained the migrated cells with deep purple color. (**b**) Data in the graphs depict that the migratory potential of chemotherapy-resistant cells (M-231/R, MCF-7/R) was substantially inhibited by CRAd treatment. The data shown above are the average of triplicate experiments (p < 0.05). (MCF-7/R is same as MCF7/DDPR; M-231/R is same as M231/DDPR).

Though the cancer cells proliferation inhibition is of great importance, cells potential to migrate is also of equal concern as it is very crucial for the cancer metastasis. Metastatic progression is among the paramount factors in cancer-related deaths. To evaluate the migration of chemotherapeutic resistant and sensitive cancer cells of breast origin in the presence of DMSO (control), and CRAd, transwell assay was performed. Micrographs were taken through phase contrast microscope (under 40x objective, at 100x magnification). Crystal violet (0.5%) stained the migrated cells in lower chambers with deep purple color. The micrographs indicated that MDR cells exposed to cisplatin or DMSO were able to migrate at different rates towards the chemoattractant (DMEM + 10% FBS) containing lower chamber of Millicells. The results showed that the migratory process was significantly inhibited in CRAd receiving cells (Fig. 6a) of both sensitive and resistant cell lines, but cessation of migration was more prominent in MDR cells (Fig. 6b).

### Reversal from EMT to MET by CRAd

**Figure 7:**
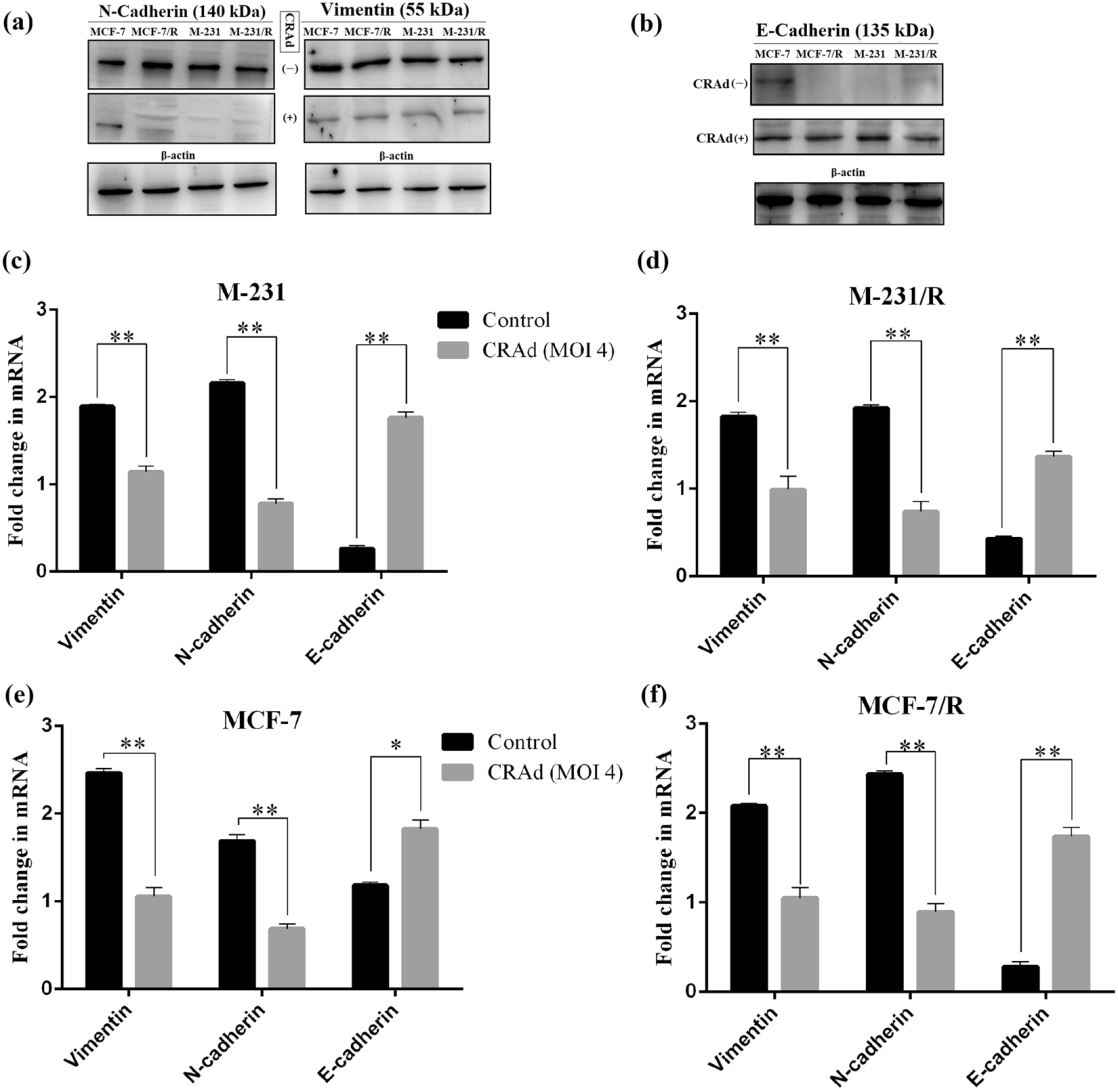
CRAd regulates cancer cell migration through EMT reversal: Breast cancer cells (MCF-7 and M-231) and their resistant sublines (MCF-7/R and M-231/R) were treated with CRAd for 48 hr at an MOI of 5 and subsequently lysed and treated for protein expression analysis via blotting. (**a**,**b**) Western blot analysis shows that CRAd treatment (+) represses mesenchymal markers, vimentin and N-cadherin, while restores epithelial marker, E-cadherin expression. Controls (un-treated cells) showed opposite trends. The data shown above are the average of triplicate experiments (p < 0.05) and blot images were cropped for clarity of the presentation. (**c**–**f**) qRT-PCR detection of mRNA levels of EMT markers in human breast cancer cell lines upon CRAd treatment. The grouped blots were cropped from different parts of the same gel. Uncropped blots are shown in the Supplementary Information (See Supplementary Data, Figs S1 and S2).

Many studies have highlighted the significant role of Epithelial-Mesenchymal Transition (EMT) markers in metastatic tumors. To find out possible mechanism behind reduction in the metastatic potential of CRAd treated breast cancer cells, we performed RT-PCR and western blot analysis of three EMT-markers (E-cadherin, N-cadherin and Vimentin). Results of this investigation indicate that in CRAd treated cells, protein levels of E-cadherin were relatively upregulated while N-cadherin and vimentin were downregulated. The breast cancer cells which did not receive any treatment showed nearly opposite trend (Fig. 7a,b). These results are consistent with those reported by Yuuri Hashimoto^29,30^ and needs further investigation. We determined mRNA levels of EMT markers (E-cadherin, N-cadherin and Vimentin) in two breast carcinoma cell lines and their chemotherapy resistant sublines by a real time RT-PCR. After normalizing *C*_T_ values for three EMT markers expression relative to NADPH levels, significant (*p* < 0.05) difference in mRNA levels were found between untreated controls and CRAd treated cells. Figure 7c–f shows that CRAd treatment increases E-cadherin expression, while downregulate the expressions of N-cadherin and Vimentin. Nearly similar results were observed in determination of protein expressions by western blotting (Fig. 7a,b).

### Cessation of tumor cell proliferation upon exposure to CRAd

**Figure 8:**
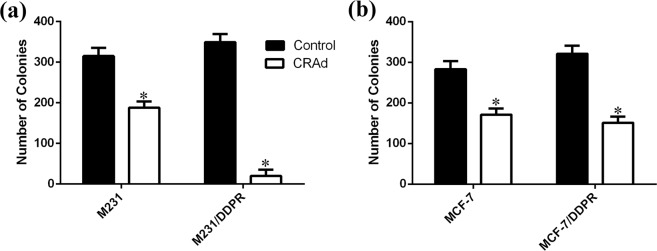
Quantitative colony formation analyses using sensitive and multidrug resistant breast cancer cells. (**a**,**b**) Breast cancer cells were treated with DMSO or CRAd for 48 hours. Cells are then washed and subsequently cultured for further 7 days. Finally, cell colonies stained with crystal violet were visualized by phase contrast microscope and counted. Each histogram shows the average ± S.D of 3 replicates from 2 individual experiments; P < 0.05) relative to control (non-treated cells).

To experimentally verify the hypothesis that CRAd-treated chemotherapeutic resistant cells which had residual metabolic activity were either dying or lost proliferation efficacy, clonogenic assay was performed. In CRAd treated cells of M231/DDPR, presence of cell colonies was almost negligible as compared to untreated group of cells (Fig. 8a,b). Significant reduction in the colony formation, as compared to control, was also observed in wells containing CRAd treated MCF7/DDPR cells. These results further confirmed the potent inhibitory effect of CRAd monotherapy against chemotherapeutic resistant breast cancer cells.

### CRAd and its co-treatment with DDP increases survival in mice with tumor xenografts

**Figure 9:**
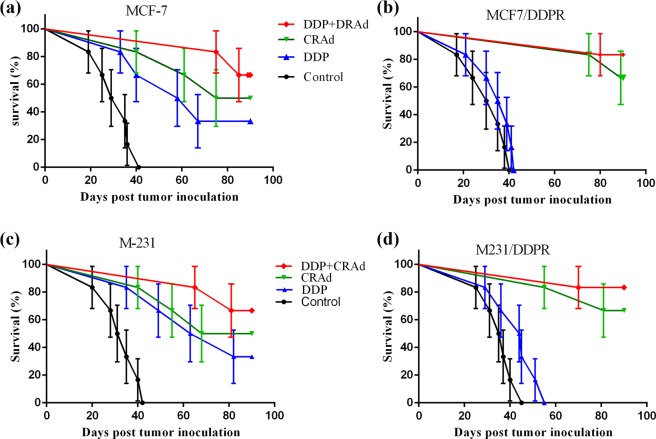
(**a**–**d**) Effect of different treatments on the survival of mice with human breast tumor xenografts. CRAd or CRAd + DDP treatments significantly raises survival in mice grafted with resistant cells, MCF-7/DDPR and M-231/DDPR (P < 0.01) but not after cisplatin monotherapy compared with control mice. In mice grafted with chemo-sensitive cells, highest survival was observed in animals which received combined therapy (DDP co-treatment with CRAd).

Our *in vivo* experiments showed nearly similar results as obtained in *in vitro* analysis. Human breast cancer cells (MCF7, MCF7/DDPR, M231, and M231/DDPR) were grafted and grown in immune-deficient mice as described previously^27^. When ~150 mm3 tumor size was achieved, mice were split into four groups having 6 animals in each group. We injected the mice bearing human breast cancer xenografts with 4 mg/kg of cisplatin intratumorally (I.T.), and 4 MOI of CRAd intraperitoneally (I.P.) in *in vivo* studies. Three mice groups received monotherapies of PBS, DDP, and CRAd, while the remaining one group was treated with combined therapy (DDP + CRAd). Animals were monitored for 90 days, and survival curves were drawn using prism 6. Figure 9a–d indicates remarkable differences in the percent survival of each treatment group. Most prominent difference was witnessed between chemotherapeutic sensitive and resistant cells. A highly promising response towards CRAd treatment was shown by resistant cells of both breast cancer cell lines (MCF/DDPR and M231/DDPR). Highest survival percentage was observed in combined treatment group which displays synergistic therapeutic activity.

When the last animal in MCF-7 xenograft control group died (day 41), 83% of CRAd, 100% of DDP + CRAd, and 66% of DDP-treated mice were alive. At the time of death of the last animal in chemotherapy resistant (MCF7/DDPR) xenograft control group (day 40), 100% of CRAd, 100% of DDP + CRAd, and 33% of DDP-treated mice were alive. Nearly similar trend in survival rates was observed among MCF-7 xenograft control and treated groups.

Mice treated with a combination therapy (DDP + CRAd) had a significantly higher survival compared to those treated with monotherapies of DDP and CRAd (P < 0.0001). Also, in resistant xenografts (mice groups grafted with MCF7/DDPR and M231/DDPR), treatment with CRAd monotherapy resulted in survivals comparable to those achieved with combination therapies (Fig. 9b,d).

## Discussion

Despite significant advancements in cancer therapy during several years, conventional treatment of breast, lung and ovarian cancers remains generally ineffective^31^. Resistance to chemotherapeutic drugs has become an inevitable event in cancer patients treatment. It necessitates the urgent hunt for novel anti-tumor therapeutic strategies. Oncolytic viruses (OVs), which kill cancer cells by exploiting cancer cell-specific vulnerabilities while sparing healthy cells, are rapidly emerging as promising weapons in the fight against cancer^32,33^. The number of OAs in clinical trials is increasing, and at present more than 12 are undergoing in different phases (I‐III) of clinical trials^33^. Resistance to chemotherapeutics may involve different factors, but overexpression of MDR1/ PGY1-gene or associated family members is often involved in multidrug resistance^13,14^. So far, none of the identified p-gp inhibitors proved clinically successful.

In our previous study^27^, we proposed that survivin regulated conditionally replicating adenovirus (Sur-CRAd) engineered in our lab could provide the desired anti-cancer goals specifically in the cases when tumors have acquired chemotherapeutic resistance. We successfully tested the hypothesis in cisplatin-resistant lung cancer cells. In this study we employed chemotherapeutic resistant cells of breast (MCF-7/DDPR, M-231/DDPR), to evaluate whether the applicability of observed outcomes was cancer type specific or whether it could be accepted more generally.

MTT assays were performed to investigate the efficacy of CRAd monotherapy on the inhibition of cell proliferation, and through the transwell assays, un-proliferating cells were observed for cell migration. Our results indicated that CRAd not only reduces cell viability but also inhibited cell migration indicating that it is highly potent anti-tumor agent. Epithelial-mesenchymal transformation is a critical biological event by which epithelial cells become motile after acquiring mesenchymal properties. In cancerous cells, it promotes the malignant, drug resistant and metastatic phenotypes. The agents which can reverse EMT are of great importance^34^. We harvested breast cancer cells pre-treated with CRAd or without prior CRAd treatment for protein expression of EMT markers. Blot results revealed significant difference in EMT markers protein levels. In untreated cancer cells, N-cadherin and vimentin proteins level was higher as compared to E-cadherin, while the cells which received pre-treatment of CRAd showed opposite results. It seems that CRAd treated cells were undergoing some kind of EMT reversal. These results concordant with a previous study which investigated the effects of an oncolytic adenovirus in lung cancer cells induced with EMT^29^. The exact mechanism of how CRAd reverses EMT still needs to elucidated.

Taken together, these insights may prove to be a timely opportunity for the application of CRAd in recurrent drug-resistant cancers. This study established that CRAd monotherapy could be a practical approach to deal with resistance issues. Further studies including clinical trials are warranted to confirm the possible use of this innovative treatment approach in clinics and move it from bench to bedside.

## Materials and Methods

### Breast Cancer Cell Lines, Adenovirus and Cell culture

The MCF-7, MDA-MB-231 cells of breast cancer were obtained from CAS-China; DDP resistant MCF-7 and MDA-MB-231 sublines, MCF-7/DDPR, M-231/DDPR, were developed by cultivating the original cells (MCF-7, M-231) in DMEM under standard conditions (37 °C, 5% CO_2_); Cells were reseeded two times/week at 3 × 10^4^ density and treated with clinically relevant doses of DDP; the drug concentration was raised from 0–76 µM in stepwise manner; subsequently cells were cloned. MCF-7/DDPR and M-231/DDPR cell clones, treated with 76 µM cisplatin dose, were selected and propagated further for 30 passages with same cisplatin sensitivity. HEK-293 cell line was obtained from Microbix Biosystems Inc. It incorporates adenoviral E1A-region for the multiplication of conditionally replicating oncolytic adenovirus, CRAd. The pEGFP-N1 vector and human full-length CAR cDNA were gift from Dr Xiang Wang (Xi’an University, Xi’an, China).

The cancer cell lines MCF, MCF-7/ADR, MCF-7/DDPR, MDA-MB-231 and M-231/DDPR, and HEK-293 cell line were cultured in Dulbecco’s modified Eagle’s medium plus 10% FBS (Gibco-BRL, HyClone); conditions for incubation were 37 °C, 5% CO2.

Survivin responsive conditionally replicating oncolytic adenovirus (CRAd) was developed in our lab. The adenoviral E1A region of CRAd was put under the control of survivin promoter; Ad-Luc expressing the firefly luciferase gene was used as a control. MCF-7/R is same as MCF7/DDPR; M-231/R is same as M231/DDPR.

### X-gal staining

Adenoviral infection efficacy was evaluated in both breast cancer cell lines and their sublines via X-gal staining. Breast cancer cells cultured in six-well plates were treated with different doses of Adβgal, and after 48 hours washed, fixed and stained according to the standard protocol (Beyotime). 0.5% glutaraldehyde was utilized to fix cells. The blue-colored product inside the cells indicates the transduced β-galactosidase gene. The number of stained cells was found by phase contrast microscope.

For more quantitative β-gal activity measurement, 10^6^ cells were cultured in a 90 mm dish for 2 days, infected for two hours with Adβgal at 4 MOI, lysed 48 hours later to measure β-galactosidase activity and normalized to the amount of protein in each sample determined by Bradford method^35^.

### *In vitro* Cytotoxicity Assay

MTT (3-(4,5-dimethylthiazol-2-yl)-2,5-diphenyltetrazolium bromide) assay was employed to evaluate the viability of the cells. Briefly, MCF-7, MCF-7/DDPR, M-231, and M-231/DDPR cells were seeded in 96 wells at a density of 1 × 10^4^ cells/180 μl/well. After 24 hours, the wells were incubated with different concentrations of cisplatin, and CRAd alone or in combination; cells were observed for 3–5 days. 10 μl/well of MTT (5 g/L) reagent was added to the cells; incubated at conditions 37 °C, 5% CO_2_ for 4 hours. Finally, absorbance at 570 nm was measured using a DNA microplate reader.

### siRNA and transfection

Chemotherapy resistant breast cancer cells (MCF7/DDPR and M231/DDPR) were chosen for siRNA mediated CAR gene knockdown. For gene knockdown, the predesigned CAR-specific siRNA, and control siRNA (negative control, NC-siRNA) sequences cloned into the ‘P-super’vector system were employed (Santa Cruz Biotechnology, Inc, Dallas, USA). The oligo sequences are as follows^37^.

CAR-siRNA: CCAAGUACCAAGUGAAGACdTdT

NC-siRNA: CACAAAAGUAUCGCGCAAGdTdT

Before transfection (puromycin-selection), cells were cultured for 24 hours in 12-well plates. Transfection using Lipofectamine reagent was done according to the instructions provided by manufacturer (Santa Cruz Biotechnology, Inc, Dallas, USA). In all experiments, 40 nM siRNAs were utilized. The success of experiment was evaluated at 48 hours after siRNA transfection via western blotting. For CAR overexpression in MCF-7 and M-231 cell lines (having low CAR expression), CAR cDNA was cloned into pEGFP-N1 via NheI and BamHI. Sequence was verified, and the Lipofectamine was used to transfect the cells according to manufacturer’s instruction.

### Semi-quantitative RT-PCR

Cisplatin sensitive and resistant breast cancer cells were cultured with DMEM (10% FBS) and subsequently using TRIzol reagent, RNA was extracted following manufacturer’s protocol (Ambion, life technologies). Isolated RNA was then converted to complimentary DNA deploying RT-reagent kit (TakaRa). The primers for CAR, ABCB1 (P-GP)^27^, ABCG2 (BCRP)^36^, ABCC1 (MRP1)^37^, ABCC2 (MRP2)^38^, E-Cadherin^39^, N-Cadherin^40^, Vimentin^41^ and β-Actin^42^ (control) are listed in Supplementary Information Table 1. The amplified products were observed via gel electrophoresis, and by quantitative real-time PCR (2−∆∆Ct method) using SYBR Green qRT-PCR premix (TaKaRa Bio, Japan). GAPDH was employed as endogenous control. The amplified products of ABCG2 (BCRP), ABCC1 (MRP1), and ABCC2 (MRP2) were observed via gel electrophoresis and the 2−∆∆Ct method. GAPDH was used as an endogenous control. The primer sequences used are listed in Table 1 given below. Gene expression level was determined by quantitative real-time PCR using SYBR Green qRT-PCR Mix (TaKaRa Bio, Japan).

**Table 1:** Primers used in RT-PCR.

Gene	Primer-F	Primer-R
CAR	CCACCTCCAAAGAGCCGTAC	ATCACAGGAATCGCACCC
ABCB1(P-GP, MDR1)	TCGTAGGAGTGTCCGTGGAT	CATTGGCGAGCCTGGTAG
ABCG2 (BCRP)	TTCGGCTTGCAACAACTATG	TCCAGACACACCACGGATAA
ABCC1 (MRP1)	CTCTATCTCTCCCGACATGACC	AGCAGACGATCCACAGCAAAA
ABCC2 (MRP2)	CCCTGCTGTTCGATATACCAATC	TCGAGAGAATCCAGAATAGGGAC
E-Cadherin	TCATGAGTGTCCCCCGGTAT	TCTTGAAGCGATTGCCCCAT
N-Cadherin	GGTGGAGGAGAAGAAGACCAG	GGCATCAGGCTCCACAGT
Vimentin	GACGCCATCAACACCGAGTT	CTTTGTCGTTGGTTAGCTGGT
β-Actin	CGCCGCCAGCTCACCATG	CACGATGGAGGGGAAGACGG

### Western blot analyses

Using roughly 10^6^ cells from each breast cancer cell line, we prepared whole cell lysates, electrophoresed them on the acrylamide gel, and then blot them onto PVDF membranes. Then, the membrane was blocked for a night. Rabbit polyclonal anti –MDR1 (P-gylcoprotein) polyclonal antibody, anti-CAR polyclonal antibody, and monoclonal antibodies for EMT markers, Vimentin, E-cadherin, N-cadherin, and mAb for β-actin were purchased from Bioss Antibodies (Bioss Inc. USA). Treatments were carried out per manufacturer’s protocol. The western blot analysis was performed as described by Amila K. Nanayakkara *et al*.^30,43^. Protein bands were visualized and photographed under transmitted ultraviolet light. Membranes were visualized using high efficiency luminescent imaging workstation (Tanon Science & Technology Co., Ltd, Shanghai, China).

### Colony formation assay

Clonogenic assay is frequently used to estimate reproductive death irradiated cells, but it can also be employed to determine the potency of many other cytotoxic agents. 4 × 10^3^ cancer cells were seeded in each well of 6 well plates for one day and incubated for two days with DMSO (control) or CRAd. After every 48 hours, cells were replenished with fresh drug-free media and allowed to grow further for seven days. Media was removed after allowed incubation period, and PBS washing was done. Cells were fixed using methanol/acetic acid 7:1. After 5 minutes, to visualize cells, staining using 0.5% crystal violet (with 25% methanol) (Alfar Aesar, MA) was done for 30 minutes. Finally, plates were washed using running water to remove crystal violet in excess and air dried. Blue dots present in confluent cell colonies indicate cells which kept growing during incubations period.

### Transwell assay

Besides development and immunity, cells migration is also very crucial for the cancer metastasis. Metastatic progression is among the crucial factors in cancer-related deaths. Migration and invasion disseminate cancer cells throughout the body. To evaluate metastatic ability of breast cancer cells, 1 × 10^5^ cells of each cell line were suspended in serum-free DMEM. These cells were plated onto 24-well Millicell chambers. To the upper Matrigel (1.0 mg/ml) coated chambers of Millicells, 5 × 10^5^ cells in serum-free medium were added. Then to the lower chamber, a chemoattractant (DMEM + 10% FBS) was added for migration analysis. Next, the chambers were incubated for 24 hours at standard conditions (37 °C in 5% CO2). After 24 h, cotton swabs were used to remove cells in the upper chambers, while the lower chamber cells were fixed using 4% paraformaldehyde. Crystal violet (0.5%) was used to stain the cells for 15 minutes. Finally, PBS washed and air-dried cells were observed and counted using a phase contrast microscope (Leica, USA) at three different fields.

### Tumor models, CRAd and cisplatin treatments

Human breast cancer cells (MCF7, MCF7/DDPR, M231, and M231/DDPR) were injected and grown in immune-deficient mice as described (27). For this study, female BALB/C nude mice (6–8 weeks old) were procured from the Chinese Academy of Sciences. Ethical guidelines provided by NIH were strictly followed during all *in vivo* experiments. The right flanks of anesthetized (ketamine hydrochloride/xylazine) mice were subcutaneously (SC) grafted with 4 × 106 breast cancer cells (MCF7, MCF7/DDPR, M231, and M231/DDPR. On the 20th day following inoculation, tumors were visible in all animals. On the basis of the respective treatments (PBS, 4 mg/kg DDP, 4 MOI of CRAd, and DDP + CRAd), tumor bearing mice were divided into four groups with six mice (n = 6) in each group. Mice were observed for three months and respective percent survival was noted and compared using prism 6. All experiments were performed following the ethical guidelines provided by NIH and in authorization by Animal Ethics Committee of Jilin University.

### Statistical analysis

To evaluate out the statistical significance, student’s t-test was employed. Statistical significance was set at p < 0.05. Prism 6 was used for the analysis of the experimental results. MS- Paint was employed to crop and label the blots. Synergism of cisplatin and CRAd was analyzed, and CI values were calculated in accordance with the Chou-Talalay method using the CompuSyn 2.0 software (ComboSyn, Inc., Paramus, NJ, USA). CI < 1 suggest synergy, while CI > 1 indicate antagonism.

## Supplementary information

Supplementary File
