# Supplementary material for: A tumor targeting oncolytic adenovirus can improve therapeutic outcomes in chemotherapy resistant metastatic human breast carcinoma
Source: Sci Rep. 2019 May 16;9:7504. doi: 10.1038/s41598-019-43668-8 (PMC6522519; doi:10.1038/s41598-019-43668-8)
Supplement: Supplementary file 1 — Supplementary File [file 41598_2019_43668_MOESM1_ESM.docx]

**Supplementary Data**

**A tumor targeting oncolytic adenovirus can improve therapeutic outcomes in chemotherapy resistant metastatic human breast carcinoma**

Sakhawat Ali, Muhammad Tahir, Aamir Ali Khan, Ma Ling, Xue Chai Chen,

Huang Yinghui ^*^

College of Life Science and Bioengineering, Beijing University of Technology

100 Ping Le Yuan, Chaoyang 100124, Beijing China.

**Supplementary Figure S1. Western blot with the gradient gel (The full length blots).** Using roughly 10^6^ cells from each breast cancer cell line, we prepared whole cell lysates, electrophoresed them on the acrylamide gel, and then blot them onto PVDF membranes. Then, the membrane was blocked for a night. Rabbit monoclonal antibodies for EMT markers, Vimentin, N-cadherin, and mAb for β-actin were purchased from Bioss Antibodies (Bioss Inc. USA). Treatments were carried out per manufacturer's protocol. The western blot analysis was performed as described by Amila K. Nanayakkara et al. [30]. Membranes were visualized using high efficiency luminescent imaging workstation (Tanon Science & Technology Co., Ltd, Shanghai, China).

**a**

**
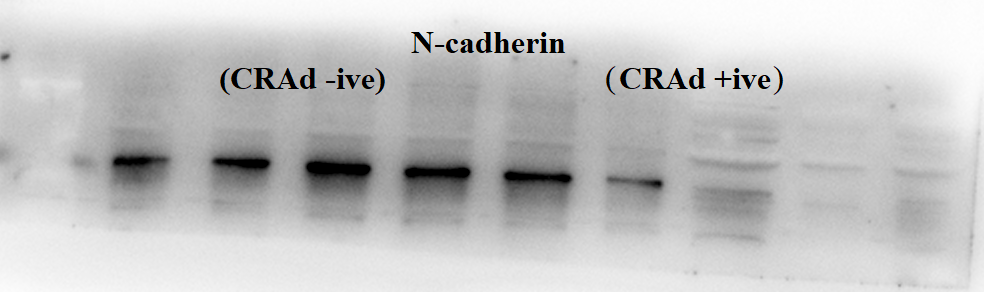
**

**b**

**
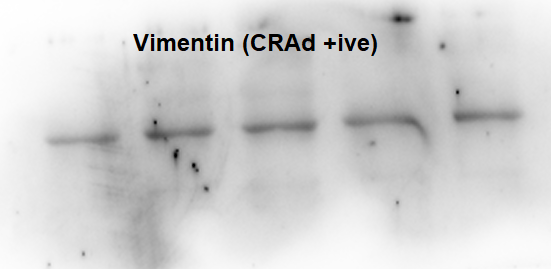

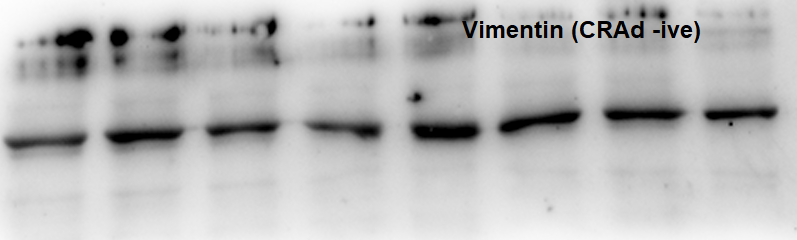
**

**c**

**
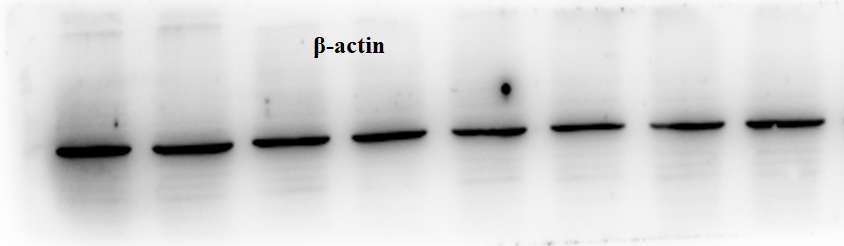
**

**Supplementary Figure S2. Full length blots blot of E-Cadherin and β-actin.** Western blot analysis shows that CRAd treatment (+) restores epithelial marker, E-cadherin expression (**a**), while E-cadherin expression in controls (un-treated cells) showed opposite trends (**b**).

**a b**

**
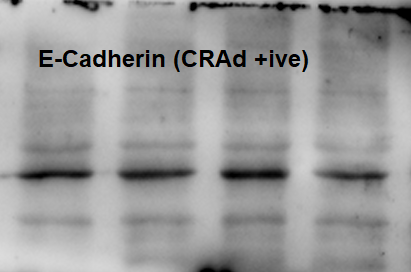

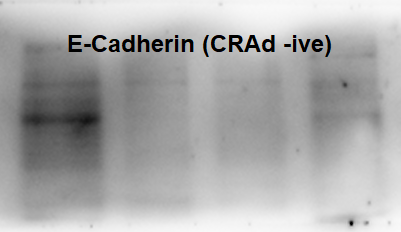
**

**c**

**
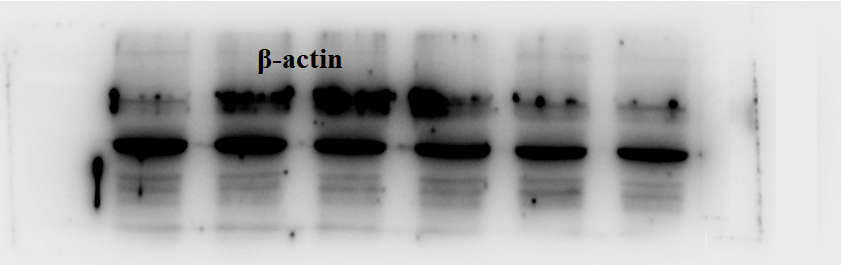
**

**Supplementary Figure S3. Full length blots of CAR in chemo sensitive breast cancer cells after over-expression and knockdown experiments.**

**a**

**
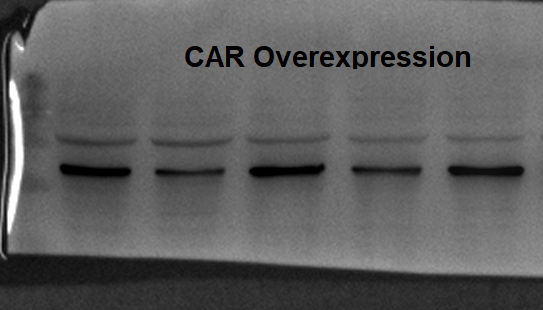

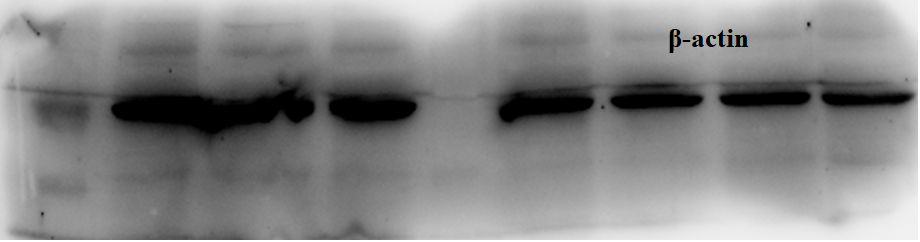
**

**b**

**
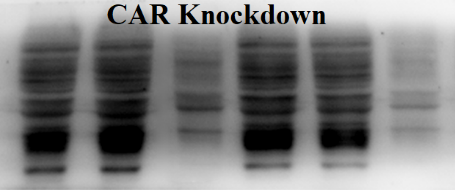

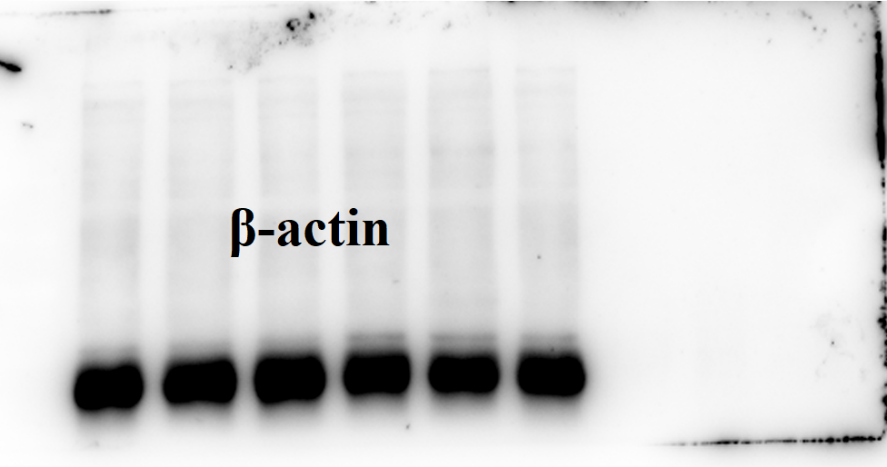
**
